# Empowering Early Recovery: The Role of Impella 5.5 in Takotsubo Cardiomyopathy Complicated by Cardiogenic Shock

**DOI:** 10.3390/jcm14176278

**Published:** 2025-09-05

**Authors:** Aarti Desai, Jose Ruiz, Anna Shapiro, Rebecca Klingbeil, Archer Martin, Rohan Goswami

**Affiliations:** 1 Division of Heart Failure and Transplantation, Mayo Clinic, Jacksonville, FL 32224, USA; desai.aarti@mayo.edu (A.D.); ruizmorales.jose@mayo.edu (J.R.); 2 Department of Anesthesia and Perioperative Medicine, Mayo Clinic, Jacksonville, FL 32224, USA; shapiro.anna@mayo.edu (A.S.); klingbeil.rebecca@mayo.edu (R.K.); martin.archer@mayo.edu (A.M.)

**Keywords:** Takotsubo cardiomyopathy, stress cardiomyopathy, cardiogenic shock, Impella, mechanical circulatory support

## Abstract

**Introduction**: Takotsubo cardiomyopathy (TCM), also known as stress cardiomyopathy or Broken Heart Syndrome, is a reversible, transient state of myocardial dyskinesis and apical ballooning. Infrequently, TCM may progress to severe life-threatening complications such as cardiogenic shock. Early mechanical circulatory support (MCS) is crucial to myocardial recovery in these cases. We present one of the first cases of TCM successfully treated with the advanced micro-axial minimally invasive Impella 5.5 with SmartAssist MCS device. **Case Presentation**: A female in her late 70s with a history of hypothyroidism, atrial fibrillation post-ablation, and cholelithiasis was referred to our facility for an elective cholecystectomy. Post-anesthesia induction with propofol 2.1 mg/kg (140 mg bolus), she became bradycardic and hypotensive, eventually leading to asystole, requiring CPR and termination of the procedure. Echocardiography revealed a left ventricular ejection fraction (LVEF) of 24% with mid-ventricular akinesis and apical ballooning with mild mitral regurgitation, suggesting the diagnosis of TCM. Cardiac catheterization showed RA 20 and mean PA 42 mmHg. Lactate was 18.7 mmol/L and LDH 1776 U/L, suggesting progressive shock. Continuous epinephrine 0.1 mcg/kg/min and norepinephrine 0.06 mcg/kg/min were titrated for BP 97/58, and she was initially supported with the Impella CP device. Despite aggressive efforts, rising LDH levels and increased vasopressor needs indicated inadequate organ perfusion, requiring an upgrade to Impella 5.5. Impella 5.5 support for 11 days led to impressive myocardial recovery, leading to reductions, and eventual discontinuation, of inotropes and vasopressors. Post-Impella 5.5 explantation, her LVEF was 59–65% and she was discharged with Mobile Cardiac Outpatient Telemetry (MCOT) monitoring for her arrhythmias and reinitiation of guideline-directed medical therapies (GDMTs) for her comorbidities. Her 2-month follow-up shows sustained LVEF greater than 45% with functional improvements. **Conclusions**: Early escalation within 24 h of Impella CP to Impella 5.5 provided stabilization of cardiometabolic shock, preventing end-organ damage, allowing recovery of native heart function while maintaining ambulatory status, and allowing for optimizing medical therapy. It presents a safe, minimally invasive, and cost-effective intervention in TCM cases refractory to GDMT or when additional time is needed for decision-making in cases presenting with CS.

## 1. Background

Takotsubo cardiomyopathy (TCM), also known as stress cardiomyopathy, is a reversible cardiomyopathy characterized by transient regional hypokinesis or akinesis and apical ballooning in the absence of acute coronary syndromes triggered by emotional or physical stress. Pathogenesis is hypothesized to be catecholamine-induced myocardial calcium overload and microvascular dysfunction, resulting in myocardial stunning or direct myocardial injury [1,2]. Though rare, around 2.4–12.4% of individuals with TCM present with profound cardiogenic shock (CS), characterized by inadequate cardiac output causing life-threatening end-organ hypoperfusion and tissue hypoxia [3]. The in-hospital mortality rate for TCM-CS patients is reported to be approximately 15% [4].

Current international guidelines recommend early initiation of mechanical circulatory support (MCS), alongside cautious use of catecholamines and inotropes [5]. The approach to treating TCM-CS varies from other causes of CS due to its dependence on the extent of left ventricular outflow tract obstruction (LVOTO). There is evidence that catecholamines, pressors, and inotropes, commonly used in CS, may worsen TCM or impede recovery due to increased LV afterload and worsening myocardial stress [3]. Consequently, the utilization of MCS is gaining popularity and demonstrating favorable results in patients with TCM at advanced stages of cardiogenic shock with or without LVOTO [4]. The Impella 5.5 with SmartAssist is a micro-axial MCS device that can provide enhanced LV unloading with flow rates up to 6 L/min and can be implanted via minimally invasive surgery via axillary cut-down, limiting complications and potentially allowing myocardial recovery [6].

We present the case of a patient in their late 70s who was admitted for an elective cholecystectomy and suffered cardiac arrest following the induction of anesthesia. The patient was diagnosed with TCM, and early mechanical circulatory support was provided using the Impella CP device, but due to increasing vasopressor needs and inadequate tissue perfusion, it was promptly exchanged for the Impella 5.5. The patient received mechanical and inotrope support for a duration of 11 days, during which time the ejection fraction showed significant improvement, reaching 59% without LV wall motion abnormalities.

## 2. Case Presentation

A female in her late 70s with a history of hypothyroidism, atrial fibrillation status post-ablation 12 years ago, and revision 3 months ago was admitted to our facility for an elective cholecystectomy for cholelithiasis. On admission, she was noted to have bradycardia (HR in her 40s) attributed to a recent ablation. Holter monitoring was scheduled by her outpatient cardiologists with the possibility of implanting a pacemaker. Her at-home medications included apixaban 5 mg BD, which we stopped prior to surgery and metoprolol tartrate 12.5 mg PRN for HR > 70 BPM. Immediately following the induction of anesthesia with propofol 2.1 mg/kg (140 mg bolus), fentanyl 100 mcg, lidocaine 100 mg, and rocuronium 50 mg, she developed bradycardia (HR in 20–30 s) and hypotension (90 systolic), eventually progressing to asystole, requiring four rounds of cardiopulmonary resuscitation (CPR), intubation, and termination of surgery.

**Figure 1:**
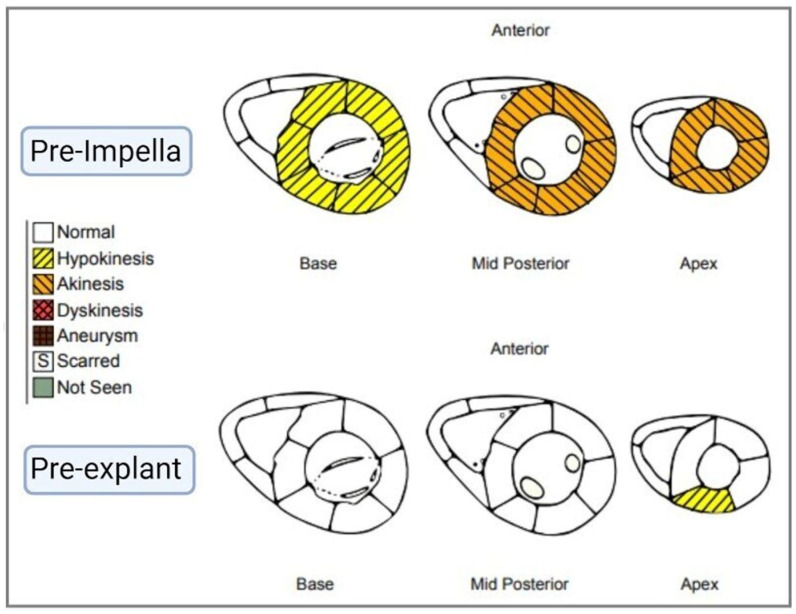
Echo-based ventricular wall motion abnormalities pre- and post-Impella placement.

Serial electrocardiograms (ECGs) showed variations between intermittent bradycardia, first-degree AV block (PR interval 360 msec), and a transient accelerated junctional rhythm, requiring the use of a temporary pacemaker. Her bedside echocardiogram revealed left ventricular ejection fraction (LVEF) 24% with anterior, mid-ventricular, and apical akinesis with mild mitral regurgitation, which led to the diagnosis of TCM, also known as stress cardiomyopathy, presenting as cardiogenic shock (RV systolic 52, RA 20, mean PA 42) (Table 1; Figure 1). Worsening shock was indicated by elevated lactate dehydrogenase (LDH) 1776 U/L, lactate 18.7 mmol/L, and central mixed venous saturation 67%. Baseline Troponin T was 149 ng/L (normal < 10 ng/L), peaking at 6 h to 585 ng/L (normal < 10 ng/L). Acute myocardial infarction was ruled out and no further troponin assays were assessed. Vasopressor infusions were initiated with epinephrine 0.1 mcg/kg/min and norepinephrine 0.06 mcg/kg/min to manage hypotension (BP 97/58). Mechanical circulatory support was provided immediately using the Impella CP (Abiomed, Danvers, MA, USA) inserted via the right femoral artery with the addition of dobutamine 5 mcg/kg/min and vasopressin 0.04 units/mL/min. Anticoagulation with bivalirudin 0.05 mg/kg/hr was initiated. The Impella CP was set to P7 with a flow rate of 3.1 L/min.

However, active bleeding at the insertion site required the transfusion of two units of packed RBCs, rising LDH levels, and increased vasopressor needs, suggesting inadequate circulatory support. The Impella CP was upgraded to the Impella 5.5 with SmartAssist the following day. The Impella 5.5 offers enhanced circulatory support, early ambulation, and potential for native myocardial recovery with flow rates reaching up to 6 L/min [6]. Under fluoroscopic and transesophageal echocardiographic guidance, the Impella CP was withdrawn after the guidewire for Impella 5.5 was pre-positioned. This allowed for rapid advancement and placement of the Impella 5.5 across the aortic valve into the LV without risk of causing aortic insufficiency. Flow was promptly initiated after confirming correct positioning, enabling seamless transition from CP to 5.5. Total time between the CP and 5.5 was less than 3 min and was bridged with background vasoactive support.

Impella 5.5 was set to P6 with a flow rate of 4 L/min. Notable improvement was evident by the conclusion of day 2 showing LVEF up to 42%. This resulted in a reduction in vasopressor needs and epinephrine was weaned off. Sustained LVEF allowed norepinephrine to be weaned off over the next 4 days. The patient was extubated on day 5.

The next 6 days showed notable improvement in LV wall contractility on echocardiography with EF up to 65% and Impella 5.5 flow was progressively reduced to 3.8 L/min by day 10 and 1.8 L/min on day 11. The Impella 5.5 was removed on day 11 and dobutamine was increased to 7.5 mcg/kg/min for post-anesthesia support. The patient continued to show stable hemodynamics without a decline in blood pressure or worsening renal function, and she was weaned off dobutamine and vasopressin over the next 2 days.

Due to acute decompensation and TCM, she had subsequent complications from volume overload and end-organ hypoperfusion, evidenced by shock liver with elevated LFTs, moderate pleural effusion, and incident Gram-positive bacteremia within her first week of admission, in addition to acute anemia. Her progression of cardiometabolic shock and end-organ hypoperfusion was mitigated by the use of the Impella 5.5. Her infection was successfully treated with vancomycin, and anemia with a total of eight packed red cell transfusions during her hospital course. The ability to maintain adequate perfusion throughout her Impella 5.5 support duration allowed the clinical team time to assess and manage each complication as it arose.

To assess the need for surgical management for her cholecystitis, a pre-discharge abdominal CT was performed. To assess the need for surgical management for her cholecystitis, a pre-discharge abdominal CT was performed. It showed small-to-moderate pericardial effusion and pericardial enhancement, causing concern of acute pericarditis, without hemodynamic compromise. The patient was discharged on colchicine 0.6 mg daily for three months with an NSAID taper of ibuprofen 600 mg TID for four weeks. She was also given the Mobile Cardiac Outpatient Telemetry (MCOT) to monitor her rhythm irregularities, and her at-home medications, apixaban 5 mg BID and metoprolol 25 mg BID, were re-initiated.

A 2-month outpatient follow-up, the echocardiogram showed sustained myocardial recovery with LVEF 59% and moderate mitral regurgitation. RV systolic pressure was 25 mmHg, and RA pressure was 5 mmHg. Atrial fibrillation seen on the prior ECG had resolved.

## 3. Discussion

TCM, or stress cardiomyopathy, is largely considered to be a state of temporary myocardial dysfunction requiring only supportive treatment; however, the severity of the presenting cardiogenic shock can be significant. Our case, with an ejection fraction of 24%, and profound vasoactive support needs, demonstrated the need for temporary mechanical circulatory support. We present this case to signify the benefits of early MCS initiation in CS cases of unknown etiology or when additional time is needed for further evaluation and management while providing sufficient circulatory support to prevent end-organ hypoperfusion. Our case demonstrates the successful use of inotropes and pressors along with a high-flow MCS device to achieve sufficient LV unloading and allow for myocardial recovery. To the best of our knowledge, this case represents one of the first few cases of TCM-CS successfully treated with the Impella 5.5 device [4].

### 3.1. Presentation and Diagnostic Criteria for TCM

TCM is an acute, reversible form of severe heart failure, believed to arise from high cardiac output and high oxygen demand states such as pregnancy, and potentially exacerbated by estrogen decline and increased sympathetic tone [7]. In non-pregnant patients, TTS is provoked by external triggers within 1–5 days of a stressful event [8]. The International Takotsubo Registry revealed that the majority of cases stem from external triggers, with physical triggers accounting for 36.0%, emotional triggers for 27.7%, and no identifiable triggers identified in 28.5% of the cases [9]. During stress, catecholamines such as epinephrine and norepinephrine are released and hypothesized to cause direct myocardial injury and microvascular dysfunction, leading to dyskinesis of the myocardium [9]. In our case, TCM was most likely brought on by the physical and emotional stress of her upcoming cholecystectomy.

Clinical findings, blood tests, ECG, echocardiography, and cardiac catheterization may be used to diagnose TCM. Patients typically exhibit symptoms such as chest pain (seen in 75% of patients), dyspnea (seen in 50% of patients), dizziness, and syncope. Other common signs of TCM include narrow pulse pressure, tachycardia, S3 gallop, and jugular venous distension. Our patient showed deterioration after the induction of anesthesia and was observed to have signs of acute systolic heart failure and hypotension, a common presentation of TCM-CS [2].

The International Takotsubo Diagnostic criteria is extensive; however, the Revised Mayo Clinic diagnostic criteria is most frequently used and includes the following [8]:

1. Transient dyskinesis of the LV midsegments with or without apical involvement; regional wall motion abnormalities beyond a single epicardial vascular distribution.
2. Absence of obstructive coronary artery disease or acute plaque rupture.
3. New electrocardiographic abnormalities or modest troponin elevation.
4. Absence of pheochromocytoma and myocarditis.

While the most encountered ECG changes in TCM are ST elevations or depressions, T wave inversions, and tachycardia, our patient showed accelerated junctional rhythm and later bradycardia [10]. This is most likely attributed to her preexisting atrial fibrillation.

Our case also substantiates the theory suggesting that Takotsubo cardiomyopathy (TCM) is more prevalent among post-menopausal women. The study of the American and International Takotsubo Registry reveals that approximately 89% of all patients of TCM are post-menopausal females with a mean age of 65 [2]. This may be attributed to the decline in cardioprotective effects of estrogen, which at pre-menopausal levels, lead to a decreased calcium overload, diminished responsiveness to catecholamines, and reduced oxidative stress. Reduction in these cardioprotective mechanisms may lead to abnormal coronary microcirculation, resulting in a myocardial state more responsive to stress hormones, leading to TCM [1,11].

This patient’s preexisting atrial fibrillation also significantly impacts the outcomes in TCM. An international, multicenter investigation involving 387 patients diagnosed with TCM and atrial fibrillation revealed elevated mortality rates, extended hospital stays, and an increased occurrence of CS. Notably, this study comprised atrial fibrillation patients managed solely with medication, whereas our patient had undergone ablation [12]. With this in mind, we will continue to monitor the patient closely for any potential adverse outcomes.

### 3.2. Current Guidelines for Treatment of TCM

The management of TCM-CS is similar to other etiologies of CS except it considers the presence of left ventricular outflow tract obstruction (LVOTO) as determined by echocardiography. In patients presenting without LVOTO, catecholamines, inotropes, and pressors may be used cautiously. However, in patients displaying LVOTO, the use of these medications is generally avoided due to their action mimicking the pathogenesis of TCM and worsening LVOTO [3]. The international guidelines for the treatment of TCM-CS outlines the following [5]:

- Avoidance of the use of catecholamines which can mimic the pathogenesis of TCM, avoidance of milrinone which showed similar effects in pre-clinical models via an increase in cAMP, and favor the use of levosimendan, a calcium sensitizer and cardiac stimulant.
- Early initiation of MCS to reduce the need for inotrope and pressor support while allowing LV recovery and providing a window for decision-making.
- Early beta blocker therapy after hemodynamic stabilization.

In our case, LVOTO was not observed, and successful management involved vigilant use of dobutamine, vasopressin, epinephrine, and norepinephrine in conjunction with early mechanical circulatory support, resulting in adequate tissue perfusion and impressive myocardial recovery.

### 3.3. Current State of Impella 5.5 in Takotsubo Cardiomyopathy

The emerging outcome data using various Impella devices for the treatment of TCM-CS have been excellent. A multicenter meta-analysis of 16 TCM-CS patients, 87.5% female, reports an 82% survival rate with the use of Impella CP or 2.5 device with all survivors (81.3%) showing complete LV recovery [13]. In this study, atrial fibrillation was absent in all survivors in this study whereas our patient has a history of atrial fibrillation and arrhythmias [13]. In a recently published case series, Nishikawa et al. report impressive myocardial recovery in four patients with TCM-CS using the Impella 2.5 for <5 days [14]. It must be noted that these cases report the use of Impella CP, 2.5, and 5. Our case represents one of the earliest to demonstrate the overwhelmingly successful use of the advanced Impella 5.5 MCS device with multiple inotrope support in the case of extremely low ejection fraction (24%) and signs of hypoperfusion due to acute TCM-CS with arrhythmia.

### 3.4. Extended Support for Recovery After Impella 5.5

A majority of TCM-CS patients will recover; however—as shown in our case report—the significant impact of hypoperfusion in propagating multi-organ decline despite increasing vasoactive needs provides an early indication of the following: 1. MCS may be needed. 2. Cardiac recovery may be possible, but end-organ function is not as clearly defined.

The utilization of the Impella CP, as outlined, usually results in very short duration of support, <5 days, whereas in patients with ongoing use of Impella CP or 2.5 devices, the risk/benefit of hemolysis, mitral valve avulsion, and renal pigment nephropathy markedly increases [15]. This case, and other data we have previously published, highlights the safety of longer-duration support in the acute setting provided by the Impella 5.5 device—allowing for potential native heart recovery [6].

## 4. Conclusions

We demonstrate that it is safe, effective, and feasible to provide early MCS with Impella 5.5 in Takotsubo Cardiomyopathy cases refractory to vasoactive support. This approach may also be utilized in cases of cardiogenic shock with unclear etiology where additional time is crucial for decision-making. Larger studies or data from randomized clinical trials may further substantiate our findings.

## Figures and Tables

**Table 1:** Vitals and hemodynamics pre-Impella, pre-explant, and at 2-month follow-up.

	Pre-Impella 5.5	Pre-Explant	2-Month Outpatient Follow-Up
**Vitals**			
Height (cm)	170.2	170.2	170.2
Weight (kg)	65.8	64.5	59.7
Body Mass Index (BMI) (kg/cm^2^)	22.7	22.3	20.7
**Hemodynamics**			
Heart Rate (bpm)	119	61	110
Blood Pressure (mmHg)	98/70	105/55	125/72
Mean Arterial Pressure (mmHg)	79	72	90
Left Ventricular Ejection Fraction (%)	24	59	59
Right Atrial Pressure (mmHg)	20	15	5
Mean Pulmonary Artery Pressure (mmHg)	48	45	-
Pulmonary Capillary Wedge Pressure (mmHg)	19	-	-
Fick Cardiac Output (L/min)	4.4	6.6	-
Fick Cardiac Index (L/min/m^2^)	2.5	3.65	-
**Laboratory values**			
Hemoglobin (g/dL)	10.6	8.7	10.6
Platelets (10^9^/L)	228	246	654
Lactate Dehydrogenase (U/L)	1776	314	-
Mixed Venous Saturation	67	53	-
Serum Lactate (mmol/L)	18.7	1.0	-

BMI = body mass index.

## Data Availability

The raw data supporting the conclusions of this article will be made available by the authors on request.

## Abbreviations

CS	cardiogenic shock
GDMT	guideline directed medical therapy
LDH	lactate dehydrogenase
LV	left ventricle
LVEF	left ventricular ejection fraction
LVOTO	left ventricular outflow tract obstruction
MCOT	mobile cardiac outpatient telemetry
MCS	mechanical circulatory support
RV	right ventricle
TCM	takotsubo cardiomyopathy
TCM-CS	takotsubo cardiomyopathy related cardiogenic shock
